# Preemptive Treatment of Nausea and Vomiting of Pregnancy: Results of a Randomized Controlled Trial

**DOI:** 10.1155/2013/809787

**Published:** 2013-02-17

**Authors:** Caroline Maltepe, Gideon Koren

**Affiliations:** The Motherisk Program, Division of Clinical Pharmacology and Toxicology, The Hospital for Sick Children, University of Toronto, 555 University Avenue, Toronto, ON, Canada M5G 1X8

## Abstract

*Objectives*. To determine whether the initiation of treatment (preemptive treatment) before the symptoms of nausea and vomiting of pregnancy (NVP) versus when the symptoms begin can improve the outcome in patients at a high risk for recurrence of severe NVP. *Study Design*. Prospective, randomized controlled trial. *Results*. Preemptive therapy conferred a significant reduction in HG as compared to the previous pregnancy (*P* = 0.047). In the preemptive arm, there were 2.5-fold fewer cases of moderate-severe cases of NVP than those in the control group (15.4% versus 39.13%) in the first 3 weeks of NVP (*P* = 0.05). In the preemptive group, significantly more women had their NVP resolved before giving birth (78.2% versus 50%) (*P* < 0.002). *Conclusions*. Preemptive treatment with antiemetics is superior to the treatment that starts only when the symptoms have already occurred in decreasing the risk of severe forms of NVP.

## 1. Introduction

Nausea and vomiting of pregnancy (NVP) is the most common medical condition in pregnancy, affecting 50–90% of pregnant women [1]. Its extreme form, hyperemesis gravidarum (HG) occurs in 0.5–3% of pregnancies leading to dehydration, electrolyte imbalance, and hospitalization. The severe forms of NVP tend to recur in up to 80% in the same women, leading to anxiety about starting another pregnancy [1]. Severe NVP has been associated with women's request to terminate otherwise—wanted pregnancies due to symptom severity [1–5].

Nausea, vomiting, and retching associated with NVP are typically treated with different classes of antiemetics [2]. The delayed release combination of 10 mg doxylamine and 10 mg pyridoxine (Diclectin, Duchesnay, Inc.) is labeled in Canada specifically for NVP and has been used by large numbers of Canadian women [1–4, 6].

In an attempt to improve the management of NVP, in 1995 the Motherisk Program at the Hospital for Sick Children in Toronto, Ontario, initiated an NVP Counseling Helpline and developed evidence-based guidelines to optimize fluid and caloric intake, while minimizing stimuli of nausea and vomiting. In 2004 we hypothesized that the preemptive use of antiemetics in women who had experienced severe NVP in the previous pregnancy may mitigate the severe symptomatology [7] in a manner similar to other conditions, such as chemotherapy-induced nausea and vomiting, motion sickness, or cyclic vomiting [8–10]. We are not aware of any other published intervention study on the preemptive effects of antiemetic modalities on the course and severity of NVP. In 1992 Czeizel and colleagues described improvement in the first-trimester nausea and vomiting associated with multivitamin use, possibly due to the antiemetic effects of vitamin B6 [11].

The objective of the present study was to compare in a randomized control trial the effectiveness of the preemptive use of Diclectin in women who had experienced severe NVP and/or HG in their previous pregnancy, to women with a similar previous experience receiving Diclectin only on the first sign of nausea, in addition to both groups receiving intensive protocolized counseling. We hypothesized that preemptive treatment will decrease the rates of HG as compared to the control group.

## 2. Patients and Methods

**Figure 1:**
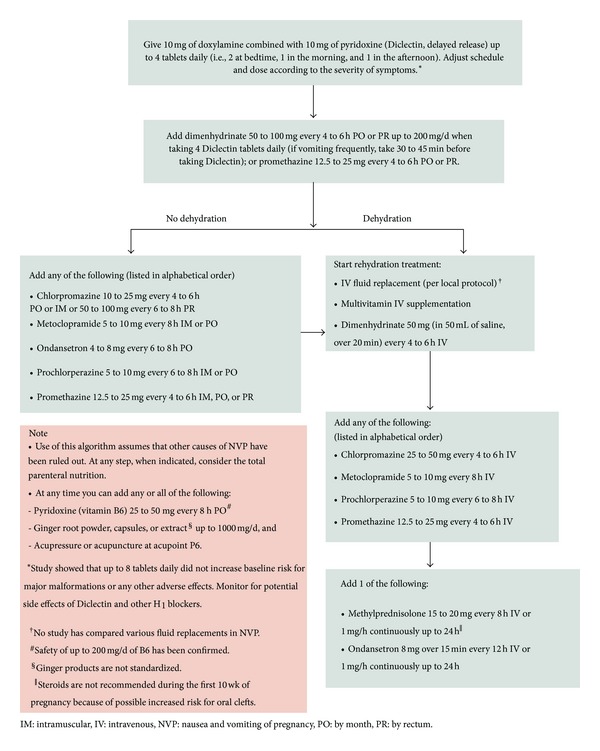
Motherisk NVP algorithm [2–4, 12].

After approval by the Hospital for Sick Children's Research Ethics Board and obtaining a written informed consent, we recruited women who were calling the Motherisk NVP Helpline for counseling. The inclusion criteria were women with a history of severe NVP in their previous pregnancy, defined either by the pregnancy-unique quantification of emesis (PUQE 24) score and/or HG, defined as severe NVP plus visits to emergency room or hospitalization for treatment of severe symptoms, always including intravenous fluids, who were currently planning a pregnancy or already pregnant but with no symptoms of NVP. The exclusion criteria were other known causes of nausea and vomiting, such as bowel diseases or gastroparesis secondary to diabetes, or inability to communicate in either English or French. The participants were randomized in a 1 : 1 ratio in a balanced 6-block design using a table of random numbers to receive Diclectin either after pregnancy was diagnosed and before any symptom of NVP emerged or to begin treatment only after the first symptom of NVP. A letter was sent to all participants' physicians reiterating their group assignment. Both participants and their healthcare providers received the Motherisk NVP treatment algorithm which is based on evidence-based counseling [2–4, 12] (see Figure 1 and Table 1). In brief, this program is based on nonpharmacological and pharmacological steps aiming to optimize fluid and caloric intake while minimizing nauseating stimuli and associated symptoms (e.g., acidity and constipation).

At the time of enrolment, all participants received counseling delineated in Table 1 and Figure 1. Women in both groups agreed to participate in a scheduled weekly and as needed telephone follow-up counseling and were reminded to contact the study coordinator as soon as they conceived and when NVP symptoms began. All participants had e-mail access to the study coordinator and all correspondence was printed and attached to their study files.

The preemptive arm started with 2 tablets of Diclectin at bedtime upon the recognition of their pregnancy, with gradual increase of their dose if NVP symptoms escalated. The control arm started with 2 tablets of Diclectin at bedtime on the first day of NVP symptoms with gradual increase of their dose if their symptoms escalated. 

With each scheduled and as needed follow-up visits, all patients received frequent additional counseling on symptom management, pharmacological and nonpharmacological, and treatment for concurrent conditions detailed in Table 1 and Figure 1. Women in both groups suffering from symptoms of gastroesophageal reflux (typically starting between 6–8 weeks of gestation) were advised, in coordination with their physicians, to take either H2 blockers or proton pump inhibitors [13–15]. 

The severity of NVP symptoms during the randomized clinical trial was measured by the validated PUQE-24 and WB scores at enrolment and at each follow-up call. The PUQE score has been validated using a large number of clinical endpoints [16–18]. The PUQE-24 score is calculated by adding the values of each of the 3 symptoms: (1) how long has the woman felt nauseated in the last 24 hours, (2) the number of episodes of vomiting in the last 24 hours, and (3) how many bouts of retching has the woman had in the last 24 hours [18] (Table 2). The WB score is a self-report of overall physical and psychological health in the 24 hours, with scores ranging from 0 (worst possible) to 10 (best possible), when compared with the woman's healthy prepregnancy state [18]. 

The severity of NVP symptoms was evaluated in several ways:

1. changes in rates of hyperemesis gravidarum (HG) as compared to the previous pregnancy for the same woman: HG was defined as severe NVP plus visits to emergency room or hospitalization for treatment of severe symptoms, always including intravenous fluids;
2. rates of resolution of NVP symptoms before the end of pregnancy and gestational age at resolution;
3. the highest PUQE score for each patient during the first 3 weeks of NVP;
4. the area under the PUQE-time curve during the first 12 weeks of gestation; later on, PUQE measures were not as commonly done to allow rigorous calculation of AUC.

The primary endpoint of the trial was the change in rates of HG in the index pregnancy compared to that observed in the previous pregnancy of the same woman.

We collected clinical characteristics, including height, weight, and BMI, past and present medical conditions, symptoms and treatments. 

## 3. Statistical Analysis

Baseline characteristics, rates of medical conditions, and measures of effectiveness were compared between the two study arms using either the Fischer exact test or Chi square (for proportions), Student's *t*-test for unpaired data, or the Mann Whitney *U* test for data that were not normally distributed. The area under the PUQE-time curve was calculated by the trapezoid rule using the patients' reported PUQE scores. Rates of changes in the primary endpoint were compared between the two arms with Fisher's exact test. Both one- and two-sided Fisher exact test were tested.

Twenty-five women per group were estimated to be necessary to identify a 40% reduction in the rate of HG with 80% power and an alpha of 0.05. The effect size of 40% was based upon the results of our previous study [7]. To account for loss to follow-up, 30 patients were randomized to each group. 

## 4. Results

A total of 31 women were randomized to the preemptive arm of the study and 29 to the control arm. One patient in the preemptive arm was excluded due to insufficient data on NVP symptoms during the first trimester (last contact with the patient was at 5.29 weeks of gestation), leaving a total of 30 evaluable preemptive cases and 29 controls. The two groups had similar baseline characteristics, as well as similar distribution of acid reflux, low iron, and motion sickness (self-reported). Women in the preemptive group tended to have more HG in their previous pregnancy than the controls, but this was not statistically significant (*P* = 0.07). Control women reported more depressive/anxiety symptoms and more headaches (Table 3). The mean (SD) gestational age (weeks) when NVP symptoms began in the preemptive group was 5.30 (SD = 1.02) in the preemptive group and 5.45 (1.88) among the controls (N.S.). Dose escalations of Diclectin were needed in all cases of both groups.

The preemptive use of Diclectin conferred a 43.3% reduction in HG between the previous pregnancy (19/30) and the present one (6/30), as compared to 17.2% reduction in the control group (from 11/29 to 6/29). The difference in the proportion of improvement between the groups was significant (*P* = 0.047, two-sided Fisher exact test). In the preemptive therapy group, there were 70% fewer cases of moderate- severe NVP (PUQE ≥ 11) as compared to the control group {4/26  (15.4%)  versus  9/23  (39.13%)} during the 3 first weeks of NVP (*P* < 0.04) (Table 4). Overall, the area under the PUQE-time curve till the end of the first 12 gestational weeks was not different between the groups (Table 4). In the preemptive group significantly more women had their NVP resolved before labor {18/23 (78.2%) as compared to the control group 11/22 (50%)} (*P* < 0.002) (Table 4). The median gestational age at the resolution of NVP symptoms was 26 weeks in the preemptive group and 33 weeks in the control group (*P* = 0.18). The mean (SD) dose of Diclectin over the period of therapy of NVP was not different between the groups {preemptive group 0.65 mg/kg/d (SD = 0.23), control group 0.56 mg/kg/d (0.24)}. The dose of Diclectin ranged from 2 to 9 tablets in both arms {mean preemptive group 0.65 mg/kg/d (0.23), control group 0.56 mg/kg/d (0.24)}.

There was a significant negative correlation between peak PUQE scores and their WB score among participants (*r* = −0.49, *P* < 0.01). Women whose peak PUQE scores were in the severe range [13, 14, 19] had median WB score of 1.5/10, those with moderate PUQE scores [7–12] had median WB scores of 5/10, and those with mild symptoms (PUQE < or equal to 6) had median WB scores of 7.5/10.

## 5. Discussion

Our results suggest, using several measures of symptom severity, that the preemptive use of Diclectin mitigated symptoms of severe NVP, as compared to the control group. Significantly less women receiving antiemetics preemptively experienced HG, and significantly more women in the preemptive group were symptom-free before the end of pregnancy, as compared to the control group. On average, women in the preemptive group needed similar doses of Diclectin to those of the control group; synthesizing these results, it appears that the preemptive use may allow early prevention of a vicious cycle of nausea and vomiting, possibly by preventing metabolic derangement and dehydration. 

Women with NVP commonly report feeling unsupported by the medical community [20–22]. Due to fears of teratogenicity, many physicians and pharmacists are hesitant to prescribe antiemetics to pregnant women, and even when they do, they often opt to use minimal doses rather than minimally-effective doses. Moreover, many physicians do not recognize the need to individualize therapy as per women's specific symptomatology. For example, large numbers of women exhibit symptoms of gastroesophageal reflux/indigestion, which are associated with more severe forms of NVP/HG and react favorably to H2 blockers or proton pump inhibitors [13–15]. 

Acknowledging this void, in 1995 The Motherisk program established the first helpline dedicated to NVP, to ensure that women receive personalized evidence-based counseling. Through our experience with the NVP Helpline, we have realized unmet needs of women after experiencing severe forms of NVP, including HG. Typically, these women exhibit high levels of anxiety and great fear that they would repeat a similar adverse experience [1, 3, 7]. 

The idea of using preemptive antiemetics for NVP stemmed from proven effectiveness of similar approaches in chemotherapy-induced nausea and vomiting, motion sickness, and cyclic vomiting [8–10]. The present study aimed to test the hypothesis that preemptive use of antiemetics can mitigate the symptoms in women who have experienced severe NVP in their previous pregnancy. 

In a prospective, nonrandomized study, 25 women who reported severe symptoms of NVP with or without HG in their previous pregnancy were recruited and counseled to commence the use of antiemetics as soon as they became aware of the present pregnancy and no later than the beginning of symptoms. They were followed up prospectively through the index pregnancy for symptoms of NVP and frequently counseled as to how to modify antiemetic doses based on symptoms. The comparison group consisted of randomly selected women also counseled by us for NVP, who had also had severe NVP in the previous pregnancy but who did not call before a planned pregnancy and thus could not be offered a preemptive therapy. The recruited women commenced the preemptive drug therapy for NVP before conception or up to 7 weeks' gestation, before the appearance of NVP symptoms. In comparison to the previous pregnancy, only eight of these 18 women experienced an HG again in the index pregnancy (*P* = 0.01). In the comparison group (*n* = 35), symptoms in the index pregnancy remained severe in 28 cases (80%), decreased to moderate in six (16.6%), and decreased to mild in five cases (13.9%). The preemptive group improved significantly compared to the control group (*P* = 0.01). However, the two groups did not differ only in preemptive use of antiemetics, but also in the intensity and content of counseling, which was far superior in the preemptive prospective group. Moreover, patients could be treated with any antiemetic drug [7].

Unlike our previous study [7], we ensured that both arms of the study (those with and without preemptive use) receive identical counseling from the NVP Helpline counselors. We have selected the combination of delayed release doxylamine and pyridoxine (Diclectin), as its fetal safety has been proven in over 200,000 pregnant women [1–4, 12, 19], and its effectiveness has been documented (originally in the 1970s, and reaffirmed in a recent randomized, blinded placebo control study) [23].

It is conceivable that, by preemptively mitigating nausea and vomiting, this approach prevents the slippery slope of metabolic effects, including starvation, dehydration, acidosis, and electrolyte imbalance [24–26]. Moreover, it is possible that it also prevents the conditioning of severe nausea and vomiting on subsequent symptoms, which is well described in chemotherapy-induced nausea and vomiting [25] and in chronic pain [27]. 

The women in the preemptive group tended to have more common HG in the previous pregnancy, making them potentially more recalcitrant to antiemetic therapy, yet they had significantly more reduction in the recurrence of HG in the present pregnancy. The control women reported more common headache and depressive/anxious symptoms in the index pregnancy. These may reflect more severe NVP symptoms due to less effective treatment. Depression by itself has not been shown to increase the likelihood of more common or more severe NVP [28] and headaches are not uncommon in severe forms of NVP. The significantly longer time till resolution of NVP in the control group and their higher likelihood of HG as compared to their previous pregnancy are consistent with our findings of lower quality of life among women with higher PUQE scores.

This study documents the effectiveness of preemptive treatment of NVP in women who had experienced severe NVP in a previous pregnancy. This study has been extremely challenging in terms of recruitment and execution. Women had to have experienced severe NVP and/or HG in their previous pregnancy, agree to start preemptive antiemetic use, and participate in an intensive counseling and follow-up program. The women in both groups received a mean of 8 follow-up calls. Moreover, the physician caring for the woman had to be a part of the healthcare team. As evidenced by the characteristics of the women, they indeed represented the severe end of this condition. 

Beyond the value of this specific protocol, our study highlights the need for personalized clinical approach to NVP, addressing the specific needs of women and the impact on their daily life. Counseling on dietary strategies, nonpharmacological and pharmacological treatment options, as well as treatment of concurrent conditions is essential for optimal management. Although the old approach that NVP may be a psychosomatic condition is fortunately not practiced anymore by most healthcare professionals, this condition is still being trivialized by many physicians, with women commonly feeling abandoned [20–22]. It is beneficial for women to receive early treatment to help reduce the severity of symptoms in future pregnancies, hopefully preventing hospitalization and improving quality of life.

This study was performed in a unique clinic that is focused on the management of NVP. As such, we are contacted by women who had experienced severe NVP in their previous pregnancy and who approach us before symptoms started in the present pregnancy. A strength of the study is the standardization of all other aspects of nonpharmacological management, guided by evidence-based counseling. The PUQE score is validated against physical signs of NVP, including ability to swallow pills, urine output, and need for hospitalization [16, 17]. The study has a few limitations that should be acknowledged: because randomizing women who had had severe NVP in the previous pregnancy to receive placebo was not felt to be ethically acceptable by the research team, this was not a blinded study due to the ethical concerns using placebo in early pregnancy. However, both arms had identical numbers of follow-up encounters, strongly suggesting that lack of blinding did not result in investigators' bias toward one arm or another. This study is a foundation for future studies and discussion on the topic. If allowed by the IRB, future studies may try to randomize women to placebo during the first part of the trial, before symptoms emerge. The study focused on the very severe end of the NVP spectrum, evidenced by the rates of HG and the late gestational age at which the symptoms resolved. Hence, the generalizability of this study to milder forms of NVP will need to be addressed in future studies. However, it is the severely affected pregnant women, whose burden of illness has been poorly managed by the medical community. Despite conducting power calculations based on the primary endpoint, the total randomized numbers were relatively small, which may result in type 1 error in the difference of baseline characteristics between the two arms.

In conclusion, our study documents that the preemptive use of the delayed combination of doxylamine-pyridoxine among women who had experienced severe NVP in the previous pregnancy can mitigate symptom severity. More studies are needed to investigate the preemptive effectiveness of other antiemetic modalities, as well as the effectiveness in less severe cases of NVP.

## Figures and Tables

**Table 1:** Symptom management protocol for NVP used in this study [2–4, 11, 13].

Dietary	Lifestyle
(i) Try eating smaller portions (even spoonful or handful), every 1-2 hrs (ii) Dry, salty, bland, and soft foods may help relieve symptoms (iii) Add a source of protein or its alternates to all meals and snacks (e.g., nuts, seeds, beans, dairy, and nut butters) (iv) Keep solids and liquids separate by drinking your fluids 20 to 30 minutes before and after you eat (v) Fluid intake should be 2 liters per day (8 cups) (vi) To help increase your fluid intake and to prevent getting dehydrated, you may also add electrolytes (e.g., sports drinks, vitamin water or coconut water) (vii) Colder fluid intake, such as slushies, popsicles and ice chips will help maintain hydration (viii) To minimize bitter or metallic taste, try adding candies, gums, or lozenges and consume colder fluids (ix) Liquid nutritional/protein products (or bars) may be added for extra nutrients	(i) To minimize heightened sense of smell, try sniffing lemons, limes, or oranges. You may also ventilate the area, have someone else cook if possible, and instead of hot foods, try to consume room temperature/cold meals or snacks (ii) It may be helpful, if you have a lot of saliva, it may be helpful to spit it out and to do frequent mouth washing (iii) Try not to brush your teeth after eating (iv) Get plenty of sleep and rest, try not to get overly tired (v) Avoid an empty stomach or feelings of hunger (vi) When rising, try to get up slowly and if possible, snack beforehand (vii) Try not to lie down after meals (viii) If possible, ask for help from family members or friends

Others	

(i) If you have low iron levels, try splitting the prenatal vitamin (take one half in the morning and one half in the evening) for tolerability.	
If your iron level is normal, try switching to a children's vitamin or a multivitamin with lower iron and added folic acid; resume with	
prenatal vitamin after 12 weeks	
(ii) For constipation, try to increase dietary fiber, such as psyllium, whole grains, fruits; and if needed, try adding a stool softener	
(e.g., docusate sodium)	
(iii) For symptoms of acidity, such as belching, burping, lump at the back of throat, burning, indigestion, and reflux, treatment	
with antacids, H2 blockers or PPIs may be suggested. Try not to overeat or leave your stomach empty. Try to sleep elevated to help	
reduce acid symptoms. Also, screening for Helicobacter pylori may be beneficial, as it has been associated with severe NVP and HG	
(iv) For symptoms of gas and/or bloating, monitoring diet and switching to lactose-free may help, and if needed, try adding an	
anti-flatulent agent (e.g., simethicone)	

**Table 2:** The Motherisk Pregnancy Unique Quantification of Emesis (PUQE-24) 24 hrs scale [18].

(1) In the last 24 hours, for how long have you felt nauseated or sick at your stomach?	Not at all (1)	1 hour or less (2)	2-3 hours (3)	4–6 hours (4)	More than 6 hours (5)	No symptoms: 3 Mild: ≤6 Moderate: 7–12
(2) In the last 24 hours, have you vomited or thrown up?	7 or more times (5)	5-6 (4)	3-4 (3)	1-2 (2)	I did not throw up (1)	Severe: 13–15
(3) In the last 24 hours, how many times have you had retching or dry heaves without bringing anything up?	No time (1)	1-2 (2)	3-4 (3)	5-6 (4)	7 or more (5)	Total score:* — *

How many hours have you slept out of 24 hours?						
Why?* — *						
On a scale of 0 to 10, how would you rate your well-being?						
*0 (worst possible)–10 (the best you felt before pregnancy) *						
Can you tell me what causes you to feel that way?* — *						

**Table 3:** Characteristics of the women in the 2 groups.

	Preemptive* (*n* = 30)	Control* (*n* = 29)	*P*
Mean age year (SD)	32.2 (4.7)	31.3 (3.2)	0.37
BMI (SD)	25.2 (5.7)	27.3 (6.6)	0.2
Some associated medical conditions			
Motion sickness	7	4	N.S.
Acid reflux/indigestion	23	27	N.S.
Depression/anxiety	8	16	0.04
Low iron/anemia	15	9	N.S.
Headaches/migraines	5	17	0.01
HG in previous pregnancy	20	11	0.07

*Mean # of followup per patient in each group: 8 (SD 1.8).

**Table 4:** Comparison of effectiveness between the two arms.

	Preemptive (*n* = 30)	Controls (*n* = 29)	*P*
Rates (%) of PUQE ≥ 11 in first 3 weeks of NVP	4 (15.4%) (*n* = 26)	9 (39.13%) (*n* = 23)	*P* < 0.04
AUC of PUQE × Time in 1st trimester (SD)	6.38 (1.58) (*n* = 25)	6.11 (2.24) (*n* = 22)	NS
NVP resolved before labor	18/23 (78.2%)	11/22 (50%)	<0.002
Gest. age when NVP resolved (median wk)	26	33	0.18
Distribution of HG in previous versus this pregnancy*			
HG in previous pregnancy	19	11	0.047*
HG in present pregnancy	6	6	
Mean daily dose of Diclectin {mg/kg (SD)}	0.65 (0.23)	0.56 (0.24)	0.2
Mean gestational age (in weeks) when NVP symptoms began (SD)	5.30 (1.02)	5.45 (1.88)	
Mean start of preemptive therapy (SD)	3.8 (0.98)		

AUC = Area under the curve.

## Abbreviations

HG: HyperemesisGravidarum
NVP: Nausea andVomiting of Pregnancy
PUQE: Pregnancy-Unique Quantification of Emesis.
